# Notch Signaling Induced by Endoplasmic Reticulum Stress Regulates Cumulus-Oocyte Complex Expansion in Polycystic Ovary Syndrome

**DOI:** 10.3390/biom12081037

**Published:** 2022-07-27

**Authors:** Hiroshi Koike, Miyuki Harada, Akari Kusamoto, Chisato Kunitomi, Zixin Xu, Tsurugi Tanaka, Yoko Urata, Emi Nose, Nozomi Takahashi, Osamu Wada-Hiraike, Yasushi Hirota, Kaori Koga, Yutaka Osuga

**Affiliations:** Department of Obstetrics and Gynecology, Faculty of Medicine, The University of Tokyo, 7-3-1, Hongo, Bunkyo, Tokyo 113-8655, Japan; hiroshikoike-tky@umin.ac.jp (H.K.); kusamoto.a77@gmail.com (A.K.); appletree0forever@hotmail.co.jp (C.K.); xuzixin0128@gmail.com (Z.X.); tsurugitanaka@gmail.com (T.T.); usapin79@hotmail.com (Y.U.); eminose0624ass@yahoo.co.jp (E.N.); nozotakahashi_plw@yahoo.co.jp (N.T.); osamu.hiraike@gmail.com (O.W.-H.); yasushi.hirota@gmail.com (Y.H.); kawotantky@gmail.com (K.K.); yutakaos-tky@umin.ac.jp (Y.O.)

**Keywords:** cumulus-oocyte complex expansion, endoplasmic reticulum stress, granulosa cell, notch signaling, polycystic ovary syndrome, unfolded protein response

## Abstract

Endoplasmic reticulum (ER) stress activated in granulosa cells contributes to the pathophysiology of polycystic ovary syndrome (PCOS). In addition, recent studies have demonstrated that Notch signaling plays multiple roles in the ovary via cell-to-cell interactions. We hypothesized that ER stress activated in granulosa cells of antral follicles in PCOS induces Notch signaling in these cells, and that activated Notch signaling induces aberrant cumulus-oocyte complex (COC) expansion. Expression of Notch2 and Notch-target transcription factors was increased in granulosa cells of PCOS patients and model mice. ER stress increased expression of Notch2 and Notch-target transcription factors in cultured human granulosa-lutein cells (GLCs). Inhibition of Notch signaling abrogated ER stress-induced expression of genes associated with COC expansion in cultured human GLCs, as well as ER stress-enhanced expansion of cumulus cells in cultured murine COCs. Furthermore, inhibition of Notch signaling reduced the areas of COCs in PCOS model mice with activated ER stress in the ovary, indicating that Notch signaling regulates COC expansion in vivo. Our findings suggest that Notch2 signaling is activated in granulosa cells in PCOS and regulates COC expansion. It remains to be elucidated whether aberrant COC expansion induced by the ER stress-Notch pathway is associated with ovulatory dysfunction in PCOS patients.

## 1. Introduction

Polycystic ovary syndrome (PCOS) is the most common endocrinological and metabolic disorder in women of reproductive age, affecting 6–20% of women in this age group. However, its pathophysiology remains to be fully elucidated [1]. Endoplasmic reticulum (ER) stress is a recently recognized local factor in the follicular microenvironment, which plays diverse roles in physiological and pathological conditions in the ovary [2]. ER stress involves the accumulation of unfolded or misfolded proteins in the ER, which is incited by various physiological and pathological conditions that increase the demand for protein folding or attenuate the protein-folding capacity of the organelle [3,4]. ER stress results in activation of several signal transduction cascades, collectively termed the unfolded protein response (UPR), which affect a wide variety of cellular functions [5,6]. Previously, we demonstrated that ER stress is activated in granulosa cells of antral follicles from PCOS patients and a mouse model of this disease [7]. Activation of ER stress modulates various cellular functions, such as the production of profibrotic cytokines, induction of apoptosis, accumulation of advanced glycation end products, and induction of aryl hydrocarbon receptor, a receptor for endocrine-disrupting chemicals, thereby contributing to the pathophysiology of PCOS [7,8,9,10].

The Notch pathway is one of the most conserved signaling systems in multicellular organisms. It regulates a variety of cellular processes, including cell fate specification, cell migration, cell adhesion, cell survival, and cell death, via juxtacrine cell-to-cell interactions [11,12]. In the ovary, various studies, especially those examining the phenotypes of Notch pathway component-knockout mice, showed that Notch signaling plays multiple roles in physiological ovarian and follicular development. It regulates follicular assembly and growth, steroidogenesis, and ovarian vascular development [11]. In addition to its physiological roles, recent studies demonstrated that Notch signaling components are aberrantly expressed in the human ovary in several pathologies including PCOS, although the findings are quite limited and conflicting [13,14,15,16,17,18,19,20]. Furthermore, the mechanisms that induce aberration of Notch signaling and the downstream pathological effects of its aberration remain unclear.

Based on these findings, we hypothesized that ER stress activated in granulosa cells of antral follicles of PCOS ovaries induces Notch signaling in these cells, and that activated Notch signaling induces aberrant cumulus-oocyte complex (COC) expansion, given that Notch signaling regulates cellular function via cell-to-cell interactions. To test this hypothesis, we measured expression of Notch2 and Hes-related family bHLH transcription factor with YRPW motif 2 (Hey2) and Hes family bHLH transcription factor 1 (Hes1) in granulosa cells of antral follicles of PCOS patients and mice with dehydroepiandrosterone (DHEA)-induced PCOS. Expression of the transcription factors Hey2 and Hes1 is driven by Notch activation upon interaction with a Notch ligand. We focused on Notch2 among the four Notch receptors (Notch1–4) since it is most abundantly expressed in granulosa cells of human antral and preovulatory follicles [21]. We determined the effects of ER stress on expression of Notch2 and Hey2 in cultured human granulosa-lutein cells (GLCs). Then, we determined the intermediary role of Notch signaling in ER stress-induced expression of various genes associated with COC expansion in cultured human GLCs. We also examined the role of Notch signaling in ER stress-induced expansion of cultured mouse COCs. Finally, we determined the in vivo effects of inhibition of Notch signaling on COC expansion in a mouse model of PCOS.

## 2. Materials and Methods

### 2.1. Human Specimens

GLCs were obtained from patients undergoing oocyte retrieval for in vitro fertilization (IVF) at the University of Tokyo Hospital, Matsumoto Ladies Clinic, and Phoenix ART Clinic. The mRNA expression levels of various Notch signaling genes were examined in GLCs of 11 PCOS patients and 12 control patients. Women with PCOS were diagnosed according to the Rotterdam criteria [22]. The inclusion criteria for control patients were a normal ovulatory cycle, no endocrinological abnormalities, and a normal ovarian morphology determined by ultrasonography. Characteristics of the patients were shown in Table 1. There were no significant differences between the groups in terms of age, body mass index (BMI), and serum basal follicle-stimulating hormone (FSH) level, while serum basal luteinizing hormone (LH) level, LH/FSH ratio, and serum anti-Müllerian hormone (AMH) level were significantly higher in PCOS patients (as expected). Immunohistochemical analysis was performed of the ovaries of four PCOS patients and three control patients. Normal ovaries were obtained from women with regular menstrual cycles without hormonal treatment who underwent radical or extended hysterectomy for carcinoma of the uterine cervix or endometrium. PCOS ovaries were obtained from women with oligo- or anovulation who also underwent hysterectomy for uterine cancer. The findings of polycystic ovaries were histologically confirmed.

### 2.2. PCOS Animal Model

A well-established DHEA-induced PCOS mouse model was used in this study [7,23]. Three-week-old female Balb/c mice were obtained from Japan SLC, Inc. (Hamamatsu, Japan). To examine activation of Notch signaling in ovaries of PCOS model mice, the animals were divided into two groups. The control group (n = 7) was subcutaneously injected daily with sesame oil for 20 days. The PCOS group (n = 7) was subcutaneously injected daily with DHEA (60 mg/kg body weight; Sigma-Aldrich, St. Louis, MO, USA) for 20 days. Ovaries were collected on day 21.

To examine the effect of inhibition of Notch signaling on COC expansion after ovulatory stimulation, PCOS model mice were intraperitoneally injected with 10 IU pregnant mare serum gonadotropin (PMSG; Asuka Pharmaceutical, Tokyo, Japan) followed 48 h later by 10 IU human chorionic gonadotropin (hCG; Mochida Pharmaceutical, Tokyo, Japan). After PMSG injection, mice were injected intraperitoneally with 25 mg/kg (3,5-difluorophenylacetyl)-L-alanyl-L-2-phenylglycine *t*-butyl ester (DAPT; Peptide Institute, Inc., Osaka, Japan) (n = 4) or DMSO (n = 4) every 24 h and euthanized at 14 h after hCG injection. DAPT is a gamma-secretase inhibitor, which inhibits release of the Notch intracellular domain (NICD), an active form of Notch, thus serves as an inhibitor of Notch signaling. The dose of DAPT administered to PCOS model mice was determined according to the previous studies [24,25]. COCs were isolated from oviducts using 27-gauge needles. The areas of COCs were measured under an optical microscope (Olympus IX70 microscope; Olympus, Tokyo, Japan).

### 2.3. Follicle Isolation and COC Culture

Three-week-old female C57/BL6j mice obtained from Japan SLC, Inc. were intraperitoneally injected with 5 IU PMSG (Asuka Pharmaceutical). Forty-eight hours after the injection, whole ovaries were collected from PMSG-primed mice and placed in Leibovitz L-15 medium (Thermo Fisher Scientific, Waltham, MA, USA) supplemented with 0.1% bovine serum albumin at 37 °C. Follicles were mechanically isolated from ovaries as previously reported [26]. Under a microscope, ovaries were cut, and follicles were mechanically isolated from ovaries with 27-gauge needles. COCs were collected from preovulatory follicles punctured with 27-gauge needles and cultured in ORIGIO Sequential Fert medium (Cooper Surgical Fertility Solutions, Malov, Denmark) containing 10 IU/mL hCG (Mochida Pharmaceutical) at 37 °C in a humidified atmosphere containing 5% CO_2_. To examine COC expansion, COCs were incubated with an ER stress inducer, 2.5 µg/mL tunicamycin (Wako, Osaka, Japan), with or without 20 μM DAPT (Peptide Institute, Inc.) for 15 h. Subsequently, the areas of COCs were measured under an optical microscope (Olympus IX70 microscope, Olympus).

### 2.4. Isolation and Culture of Human GLCs

GLCs were isolated as previously reported [8,27]. Follicular fluids were centrifuged, and the pellet was resuspended in phosphate-buffered saline (PBS) containing 0.2% hyaluronidase (Sigma-Aldrich) and incubated at 37 °C for 30 min. The suspension was centrifuged again at 400 g for 30 min after being layered over Ficoll-Paque (GE Healthcare, Buckinghamshire, UK). GLCs were collected from the interface and washed with PBS. They were cultured in 12- or 48-well plates at a density of 5 × 10^5^ or 5 × 10^4^ cells/well, respectively, in Dulbecco’s modified Eagle’s medium F-12 (DMEM/F-12; Thermo Fisher Scientific) containing 10% fetal bovine serum (Sigma-Aldrich) and antibiotics (100 U/mL penicillin, 0.1 mg/mL streptomycin, and 250 ng/mL amphotericin B; Sigma-Aldrich). Prior to experiments, all GLCs were precultured for 3–5 days at 37 °C in a humidified atmosphere containing 5% CO_2_.

### 2.5. Treatment of Human GLCs

To evaluate the effect of ER stress on Notch signaling, human GLCs were preincubated with an ER stress inhibitor, 1 mg/mL tauroursodeoxycholic acid (TUDCA; Tokyo Chemical Industry Co., Tokyo, Japan), for 24 h, and then incubated with ER stress inducers, 2.5 µg/mL tunicamycin (Wako), for 24 h or 1 µM thapsigargin (Sigma-Aldrich), for 6 h. The optimal concentrations of these drugs were chosen based on previous studies using human GLCs [7,9,10]. To examine the effect of ER stress on expression of genes associated with COC expansion, human GLCs were incubated with 2.5 μg/mL tunicamycin and 50 μM DAPT in DMEM/F-12 containing 10 IU/mL hCG for 6 h. To knockdown activating transcription factor 4 (ATF4), siRNA was obtained from Dharmacon (GE Healthcare) as SMART pools: ON-TARGET plus human ATF4 (L-005125) for knockdown and ON-TARGET plus nontargeting pool (D-001810-10-20) as the negative control. GLCs were transfected with 50 nM siRNA using Lipofectamine RNAiMAX (Thermo Fisher Scientific) for 24 h. After transfection, the medium was removed, and GLCs were incubated with 2.5 μg/mL tunicamycin for 24 h.

### 2.6. RNA Extraction, Reverse Transcription, and Real-Time Quantitative PCR

The RNA was isolated from human GLCs, followed by synthesizing the cDNA template, using a SuperPrep II Cell Lysis & RT Kit for qPCR (TOYOBO, Osaka, Japan). To measure the expression levels of mRNAs, quantitative real-time PCR was performed on a Light Cycler system (Roche Diagnostics GmBH, Mannheim, Germany). The mRNA expression levels were normalized to that of glyceraldehyde-3-phosphate dehydrogenase (GAPDH), which served as an internal control. The primer sequences are shown in Table 2. The PCR conditions were as follows: 40 cycles of 98 °C for 10 s, 60 °C for 10 s, and 68 °C for 30 s. All samples were analyzed in triplicate or quadruplicate for in vitro experiments.

### 2.7. Western Blotting

GLCs were lysed in PhosphoSafe Extraction Reagent (Merck, Darmstadt, Germany) and centrifuged at 16,000× *g* for 5 min. Supernatants were recovered, and the protein concentration was measured using the Bio-Rad Protein Assay (Bio-Rad Laboratories, Hercules, CA, USA). Equivalent amounts of denatured protein were subjected to SDS-PAGE and then electrophoretically transferred to polyvinylidene difluoride membranes using the Trans-Blot Turbo Transfer System (Bio-Rad Laboratories). After blocking with 5% skim milk prepared in Tris-buffered saline containing 0.1% Tween-20 at room temperature for 1 h, membranes were probed with an anti-ATF4 (1:000, RRID: AB_2616025, Cell Signaling Technology, Danvers, MA, USA), anti-Notch2 (1:5000, RRID: AB_10693319; Cell Signaling Technology), or anti-β-actin (1:10,000, RRID: AB_476697, Sigma-Aldrich) antibody overnight at 4 °C and then incubated with secondary antibodies (antirabbit RRID: AB_2099233 or anti-mouse RRID: AB_330924, Cell Signaling Technology) at room temperature for 1 h. Images were acquired using ECL Plus western blotting detection reagents (GE Healthcare) on an ImageQuant LAS 4000 Mini luminescent image analyzer (GE Healthcare). β-actin was used as a loading control. The immunoblot procedure was repeated at least three times. Band intensity was quantified using ImageJ software (RRID: SCR_003073; National Institutes of Health, Bethesda, MD, USA) [28].

### 2.8. Histology and Immunohistochemistry

Human and mouse ovaries were fixed in 10% neutral buffered formalin, embedded in paraffin, and sectioned at a thickness of 5 μm. Sections obtained from the center of each ovary were stained with hematoxylin and eosin. The ovarian sections were immunostained with an anti-Notch2 (1:200, RRID: AB_10693319, Cell Signaling Technology), anti-Hey2 (1:500, AB_2118415; Proteintech Group, Tokyo, Japan), or anti-Hes1 (1:400, RRID: AB_1209570; Abcam, Cambridge, UK) antibody using an EnVision+ Dual Link System/HRP (DAB) Kit (Dako, Tokyo, Japan). Isotype-specific IgG served as a negative control. Antigen retrieval was performed using Target Retrieval Solution (Dako). Immunohistochemistry was performed at least three times independently using identical samples. Stained sections were examined by light microscopy using an Olympus BX50 fluorescence microscope (Olympus). ImageJ software (National Institutes of Health) was used for quantitative analysis [28].

### 2.9. Statistical Analysis

All statistical analyses were performed using JMP Pro 15 software (RRID: SCR_022199; SAS Institute Inc., Cary, NC, USA). All data are shown as means ± standard error of the mean (SEM). Data were analyzed using the Student’s *t*-test for paired comparisons and the Tukey-Kramer honest significant difference test for multiple comparisons. *p* < 0.05 was considered statistically significant.

## 3. Results

### 3.1. Notch2, Hey2, and Hes1 Are Upregulated in Granulosa Cells of PCOS Patients

**Figure 1:**
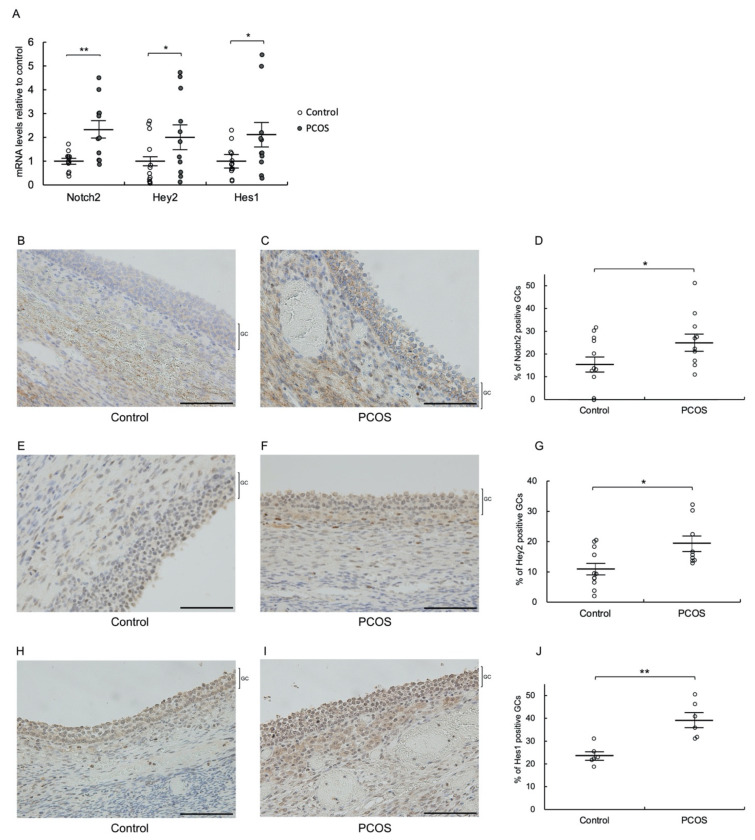
Notch2, Hey2, and Hes1 are upregulated in granulosa cells of PCOS patients. (**A**) The expression levels of Notch2, Hey2, and Hes1 mRNAs in GLCs from control (n = 12) and PCOS (n = 11) patients were measured by real-time PCR and normalized to that of GAPDH. (**B**–**J**) Immunohistochemical analysis was performed on antral follicles of ovaries from control (n = 3) and PCOS (n = 4) patients. Cross-sections of ovaries were stained with an (**B**–**D**) anti-Notch2, (**E**–**G**) anti-Hey2, or (**H**–**J**) anti-Hes1 antibody and counterstained with hematoxylin. (**D**,**G**,**J**) Quantitative analysis of immunohistochemical staining. Values represent means ± SEM, relative to the mean control value. The scale bars indicate 100 μm. * *p* < 0.05 and ** *p* < 0.01 compared with control. GC, granulosa cell layer; GCs granulosa cells.

To evaluate Notch signaling in granulosa cells of antral follicles of PCOS patients, we measured the mRNA expression levels of Notch2 and the transcription factors Hey2 and Hes1, which are activated by Notch signaling, in GLCs of PCOS patients using quantitative real-time PCR. We focused on Notch2 among the four Notch receptors (Notch1–4), and Hey2 and Hes1 among several Notch target genes, including Hey1, 2, and L and Hes1–7, since Notch2 and Hey2/Hes1 are the most abundantly expressed Notch receptor and target genes, respectively, in granulosa cells of human antral and preovulatory follicles [21]. The mRNA levels of Notch2, Hey2, and Hes1 were significantly higher in GLCs of PCOS patients than in GLCs of control patients (*p* < 0.01 for Notch2, and *p* < 0.05 for Hey2 and Hes1) (Figure 1A). Subsequently, we performed immunohistochemical analysis of ovaries from PCOS patients to confirm the protein expression of Notch signaling components. Immunoreactivity of Notch2, Hey2, and Hes1 was significantly higher in granulosa cells of antral follicles from PCOS patients than in those from control patients (*p* < 0.05 for Notch2 and Hey2, and *p* < 0.01 for Hes1) (Figure 1B–J).

### 3.2. Notch2, Hey2, and Hes1 Are Upregulated in Granulosa Cells of PCOS Model Mice

**Figure 2:**
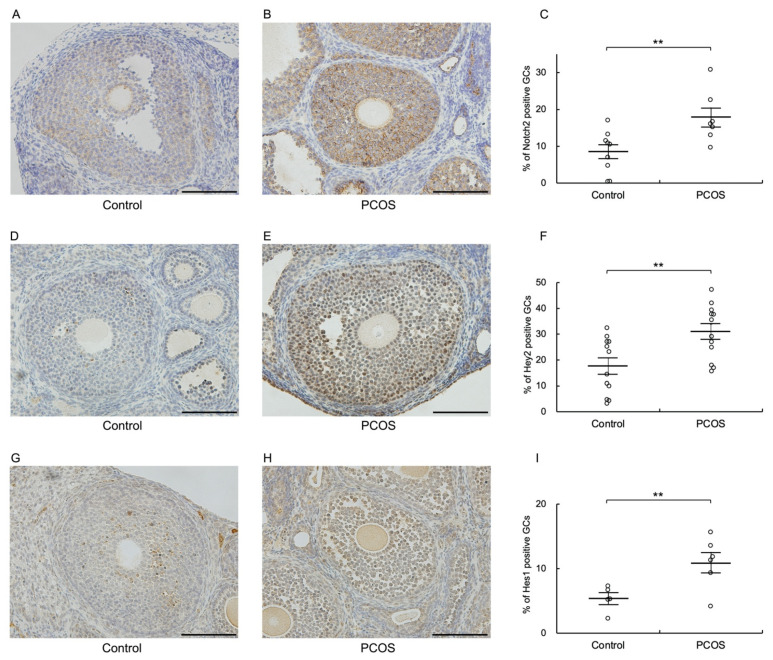
Notch2, Hey2, and Hes1 are upregulated in granulosa cells of PCOS model mice. Immunohistochemical analysis was performed on antral follicles of ovaries from control (n = 7) and PCOS (n = 7) mice. Cross-sections of ovaries were stained with an (**A**–**C**) anti-Notch2, (**D**–**F**) anti-Hey2, or (**G**–**I**) anti-Hes1 antibody and counterstained with hematoxylin. (**C**,**F**,**I**) Quantitative analysis of immunohistochemical staining. The scale bars indicate 100 μm. ** *p* < 0.01 compared with control. GCs, granulosa cells.

Next, we investigated expression of Notch2, Hey2, and Hes1 in ovaries of PCOS model mice. Immunohistochemical analysis revealed that the expression levels of Notch2, Hey2, and Hes1 were significantly higher in granulosa cells of antral follicles from PCOS model mice than in those from control mice (*p* < 0.01) (Figure 2). Together with the observations of human specimens, these findings indicate that Notch2 signaling is induced in granulosa cells of antral follicles in PCOS.

### 3.3. ER Stress Increases Notch2 and Hey2 Expression in Cultured Human GLCs

**Figure 3:**
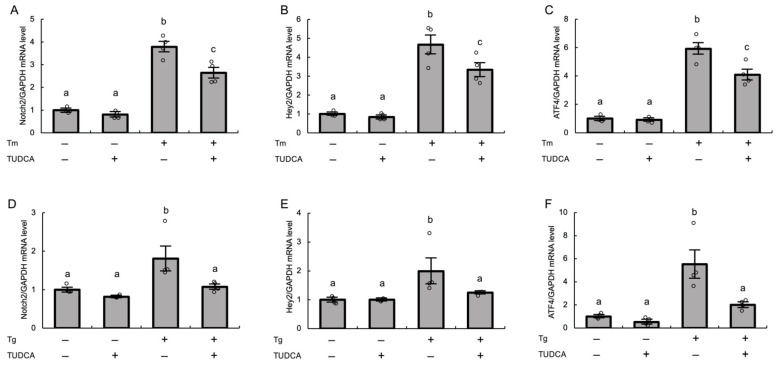
Effects of ER stress on Notch2 signaling in cultured human GLCs. After preincubation with TUDCA (1 mg/mL) for 24 h, human GLCs were treated with (**A**–**C**) tunicamycin (2.5 μg/mL) for 24 h or (**D**–**F**) thapsigargin (1 μM) for 6 h. The expression levels of (**A**,**D**) Notch2, (**B**,**E**) Hey2, and (**C**,**F**) ATF4 mRNAs were measured by real-time PCR and normalized to that of GAPDH. Values represent means ± SEM, relative to the mean control value. The letters denote significant differences between groups. Tm, tunicamycin; Tg, thapsigargin.

To determine whether ER stress, which is activated in granulosa cells of antral follicles in PCOS, is associated with induction of Notch2 signaling in these cells, we measured the mRNA expression levels of Notch2 and Hey2 following treatment with an ER stress inducer, tunicamycin or thapsigargin, in cultured human GLCs. Based on the results of the expression pattern of Notch2 signaling molecules, we examined Hey2 as a representative target gene of Notch2 signaling hereafter. Tunicamycin and thapsigargin induce ER stress in different manners: tunicamycin induces ER stress by blocking N-linked glycosylation, resulting in accumulation of misfolded proteins in the ER, while thapsigargin induces ER stress by inhibiting Ca^2+^ uptake into the ER, thereby attenuating the organelle’s protein-folding capacity [10]. Both tunicamycin and thapsigargin significantly increased the levels of Notch2 and Hey2 (*p* < 0.01), indicating that ER stress upregulates Notch2 and Hey2 in human GLCs. This was further confirmed by the finding that pretreatment with the ER stress inhibitor TUDCA significantly abrogated the positive effects of these ER stress inducers on Notch2 and Hey2 expression (Figure 3A,B,D,E). The examination of the expression level of ATF4, an UPR factor, confirmed the roles of tunicamycin and thapsigargin as ER stress inducers and the role of TUDCA as an ER stress inhibitor, respectively (Figure 3C,F). 

### 3.4. ER Stress Induces Notch2 Signaling via the ATF4 Pathway in Cultured Human GLCs

**Figure 4:**
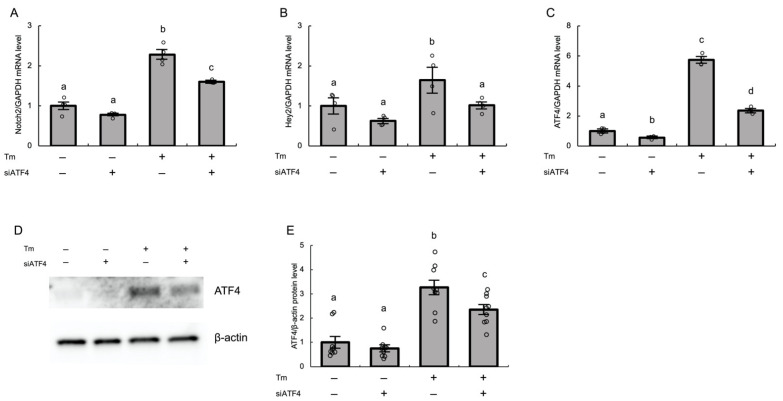
ER stress induces Notch2 signaling via the ATF4 pathway in cultured human GLCs. Human GLCs were transfected with siATF4 or negative control siRNA for 24 h and then treated with tunicamycin (2.5 μg/mL) for 24 h. The expression levels of (**A**) Notch2, (**B**) Hey2, and (**C**) ATF4 mRNAs were measured by real-time PCR and normalized to that of GAPDH. (**D**) The protein expression level of ATF4 in GLCs was analyzed by western blotting. β-Actin was used as a loading control. (**E**) Quantitative analysis of western blotting. Values represent means ± SEM, relative to the mean control value. The letters denote significant differences between groups. Tm, tunicamycin.

Among the three UPR branches activated by ER stress, several studies demonstrated an association between the UPR branch involving ATF4 and Notch signaling in various contexts [29,30,31]. Thus, we examined whether the ATF4 pathway mediates ER stress-induced upregulation of Notch2 and Hey2 in granulosa cells. We used tunicamycin as an ER stress inducer hereafter. Pretreatment of cultured human GLCs with ATF4-targeting siRNA (siATF4) significantly abrogated tunicamycin-induced expression of Notch2 and Hey2 in these cells, and concomitantly reduced ATF4 mRNA expression (*p* < 0.01) (Figure 4A,B). Knockdown of ATF4 by siRNA was confirmed by real-time PCR and western blotting (Figure 4C–E).

### 3.5. Notch2 Signaling Mediates ER Stress-Induced Expression of Various Genes Associated with COC Expansion in Cultured Human GLCs

**Figure 5:**
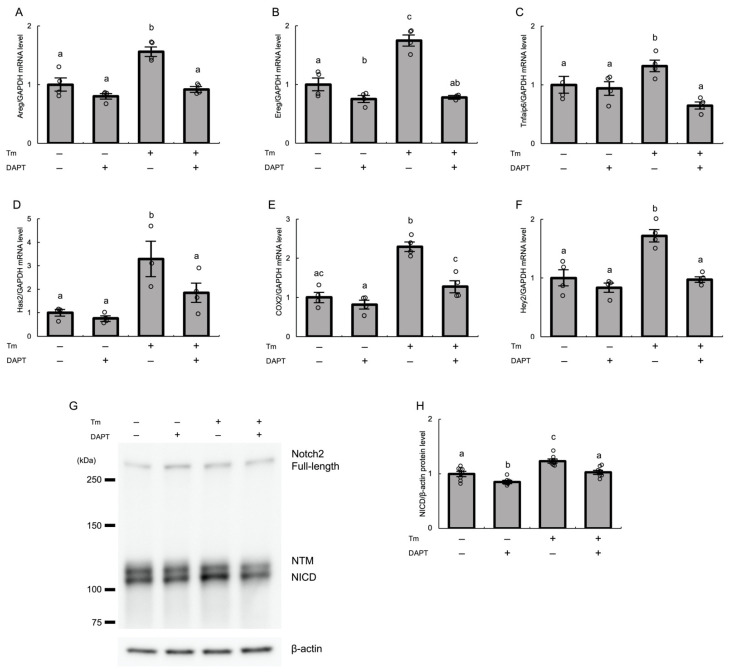
Notch2 signaling mediates ER stress-induced expression of various genes associated with COC expansion in cultured human GLCs. Human GLCs were incubated with tunicamycin (2.5 μg/mL) and DAPT (50 μM) in medium containing hCG (10 IU/mL). The expression levels of (**A**) Areg, (**B**) Ereg, (**C**) Tnfaip6, (**D**) Has2, (**E**) COX2, and (**F**) Hey2 mRNAs were measured by real-time PCR and normalized to that of GAPDH. (**G**) The protein expression level of an active form of Notch2 (Notch intracellular domain) in GLCs was analyzed by western blotting. β-Actin was used as a loading control. (**H**) Quantitative analysis of western blotting. Values represent means ± SEM, relative to the mean control value. The letters denote significant differences between groups. Tm, tunicamycin; NTM, Notch transmembrane domain; NICD, Notch intracellular domain.

To examine whether ER stress-induced Notch2 signaling is involved in expansion of cumulus cells, we first measured the mRNA levels of genes associated with COC expansion, including amphiregulin (Areg), epiregulin (Ereg), tumor necrosis factor alpha-induced protein 6 (Tnfaip6), hyaluronan synthase 2 (Has2), and cyclooxygenase 2 (COX2). Treatment with tunicamycin significantly increased the mRNA expression levels of all of these genes in cultured human GLCs (*p* < 0.01), and these effects were significantly abrogated by treatment with DAPT (*p* < 0.01 for Areg, Ereg, Tnfaip6, and COX-2, and *p* < 0.05 for Has2) (Figure 5A–E). DAPT serves as a Notch inhibitor by inhibiting gamma-secretase, which releases NICD, an active form of Notch, which in turn drives expression of Notch target genes, including Hey2. The inhibitory effect of DAPT on activation of Notch2 signaling induced by ER stress was confirmed by analysis of Hey2 mRNA expression and of protein expression of NICD, an active form of Notch2. Tunicamycin-induced Hey2 mRNA expression was abrogated by treatment with DAPT (*p* < 0.01) (Figure 5F). Furthermore, western blot analysis showed that treatment with DAPT significantly reduced the tunicamycin-induced expression of NICD in GLCs (*p* < 0.01) (Figure 5G,H).

### 3.6. Notch Signaling Mediates ER Stress-Enhanced Expansion of Cultured Murine COCs

**Figure 6:**
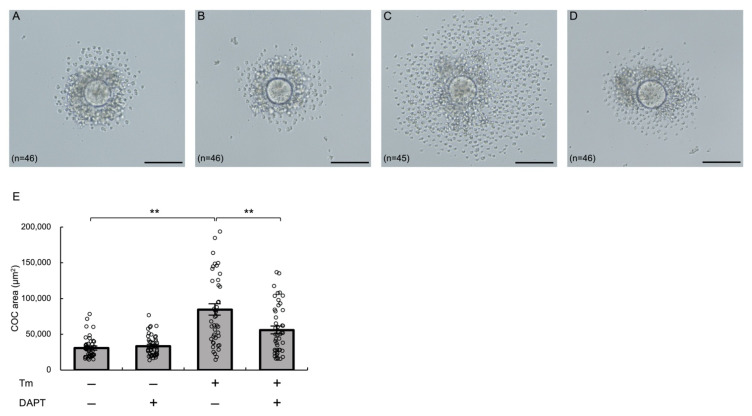
Notch signaling mediates ER stress-enhanced expansion of cultured murine COCs. COCs isolated from large antral follicles of PMSG-primed mouse ovaries were cultured in medium containing hCG (10 IU/mL) supplemented with tunicamycin (2.5 μg/mL) and/or DAPT (20 μM) for 15 h. (**A**–**D**) Representative microscopic images of COCs in each treatment group: (**A**) control, (**B**) DAPT, (**C**) tunicamycin, and (**D**) tunicamycin + DAPT. The scale bars indicate 100 μm. (**E**) Quantitative analysis of the areas of COCs. Values represent means ± SEM. ** *p* < 0.01. Tm, tunicamycin.

Next, we examined the effects of activation of ER stress and inhibition of Notch signaling on COC expansion using cultured murine COCs. COCs were obtained from preovulatory follicles of PMSG-primed mice, and the areas of COCs in each treatment group were measured under an optical microscope. Treatment with tunicamycin significantly increased the COC areas (*p* < 0.01), and this effect was significantly abrogated by treatment with DAPT (*p* < 0.01) (Figure 6). These findings suggest that Notch signaling plays an intermediary role in ER stress-enhanced expansion of COCs.

### 3.7. Notch Signaling Mediates COC Expansion in PCOS Model Mice

**Figure 7:**
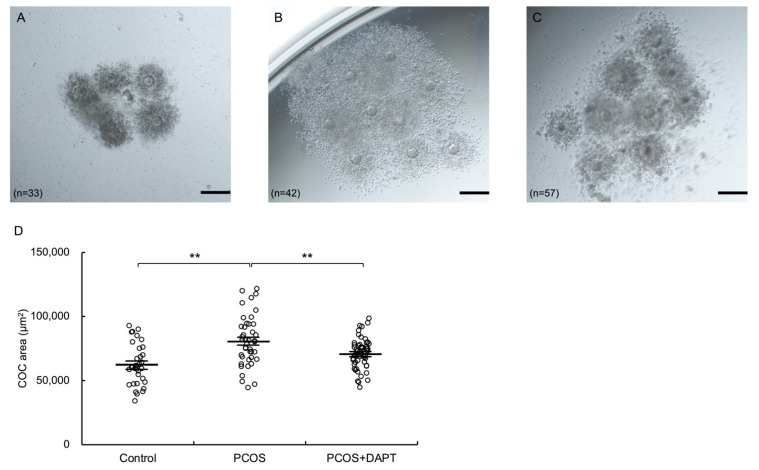
Notch signaling mediates COC expansion in PCOS model mice. Mice were injected subcutaneously with DHEA (60 mg/kg body weight) (PCOS, n = 4 or PCOS + DAPT, n = 4) or sesame oil (control, n = 4) daily for 20 days. Mice were injected intraperitoneally with 10 IU PMSG followed 48 h later with 10 IU hCG. After PMSG injection, mice were injected intraperitoneally with DAPT (25 mg/kg) (PCOS + DAPT) or DMSO (control or PCOS) every 24 h. Mice were euthanized at 14 h after hCG injection. COCs were isolated from oviducts. (**A**–**C**) Representative microscopic images of COCs harvested from each group of mice: (**A**) control, (**B**) PCOS, and (**C**) PCOS treated with DAPT. The scale bars indicate 200 μm. (**D**) Quantitative analysis of the areas of COCs. Values represent means ± SEM. ** *p* < 0.01.

Given that ER stress is activated in PCOS ovaries [7], we wondered whether COC expansion is affected in PCOS and, if so, whether Notch signaling plays a regulatory role in this process in vivo. The areas of COCs collected from oviducts after induction of superovulation followed by hCG administration were significantly larger in PCOS model mice than in control mice (*p* < 0.01) (Figure 7). Furthermore, administration of DAPT significantly reduced the COC areas of PCOS mice (*p* < 0.01). These findings suggest that Notch signaling regulates COC expansion in PCOS.

## 4. Discussion

The present study shows that expression of Notch2 and the Notch-target transcription factors Hey2 and Hes1 is higher in granulosa cells of antral follicles of PCOS patients and a mouse model of PCOS than in those of control participants and control mice, respectively. ER stress, which is activated in granulosa cells of antral follicles in PCOS, increased expression of Notch2 and Hey2 in cultured human GLCs, and this was mediated by the UPR branch involving ATF4. ER stress increased the expression of genes associated with COC expansion in cultured human GLCs and enhanced the expansion of cumulus cells in cultured murine COCs, and these effects were abrogated by inhibition of Notch signaling. Finally, COC expansion was enhanced in PCOS model mice, and inhibition of Notch signaling reduced the areas of COCs in these mice.

Expression of Notch2, Hey2, and Hes1, which are the most typical Notch receptor and target transcription factors of Notch signaling in granulosa cells of antral follicles, is increased in these cells in PCOS, indicating that Notch2 signaling is induced in PCOS. Existing findings about Notch in PCOS ovaries are quite limited and conflicting. Furthermore, most of them are based on transcriptome profiling of Notch and/or target genes, without examination of protein expression [13,14,15,16,17,18,19,20]. In addition, all existing reports about granulosa cells utilized human GLCs or cumulus cells harvested at IVF after ovarian stimulation. Bioinformatic analysis showed that Notch signaling pathways are enriched in PCOS [20]. Notch1 and Notch2 mRNAs were upregulated in GLCs/cumulus cells of PCOS patients compared with those of control participants in some reports [15,18], while other reports showed that Notch2, Notch3, and Hes1 mRNAs were downregulated [16,17,19]. The present study showed that mRNA expression of Notch2 and target transcription factors of Notch2 signaling, Hey2 and Hes1, was increased in GLCs of PCOS patients. We further demonstrated that protein expression of Notch2, Hey2, and Hes1 was increased in granulosa cells of antral follicles in PCOS patients without ovarian stimulation necessary for IVF, as well as in PCOS model mice. Together, our findings suggest that Notch2 signaling is induced in granulosa cells of antral follicles in PCOS.

Two types of ER stress inducers increased expression of Notch2 and Hey2 in human GLCs, and these effects were abrogated by pretreatment with an ER stress inhibitor. This shows that ER stress, which is activated in granulosa cells of antral follicles in PCOS, induces Notch2 signaling in these cells. ER stress activates Notch signaling in various human cells, including kidney cells, pancreatic β-cells, glioma cells, vascular endothelial cells, and coronary smooth muscle cells [29,31,32,33,34,35]. ER stress and Notch signaling are speculated to be closely associated in various tissues. Under ER stress, the sensor proteins, inositol-requiring enzyme 1 (IRE1), double-stranded RNA-activated protein kinase-like ER kinase (PERK), and activating transcription factor 6 (ATF6), activate the three branches of the UPR. Induction of a transcription factor ATF4 is involved in the UPR pathway activated by PERK [5]. Our study shows that the UPR branch involving ATF4 mediates induction of Notch2 signaling by ER stress in human GLCs. The ATF4 pathway is closely related to proinflammatory signaling in various cells including human granulosa cells [27,36,37]. Notch signaling is activated in several pathologies associated with inflammation, including nonalcoholic steatohepatitis and osteoarthritis, and contributes to their pathogenesis [38,39,40]. Taken together with the finding that the microenvironment of follicles is inflammatory in PCOS ovaries [41,42], our results suggest that it is plausible that ER stress, inflammation, and Notch signaling worsen the follicular microenvironment in PCOS in a coordinated manner.

ER stress enhanced COC expansion in vitro, which was accompanied by increased expression of various genes associated with COC expansion, and the effects were abrogated by inhibition of Notch2 signaling. Two reports have evaluated the role of ER stress in COC expansion [43,44], while no study has investigated the role of Notch in this phenomenon. The ER stress inducer thapsigargin enhances murine COC expansion and expression of related genes [43], consistent with the findings of this study. COC expansion is impaired in model rats lacking ATF4 in their ovarian tissue [44]. The findings of our in vitro study show that Notch2 signaling mediates ER stress-induced COC expansion. No previous study has examined the mechanisms that regulate ER stress-induced COC expansion and related genes. Given that Notch signaling regulates a variety of cellular processes via cell-to-cell interactions, it plausibly plays a regulatory role during COC expansion where interactions between cumulus cells change drastically. PCOS model mice treated with DAPT exhibited smaller areas of COCs than nontreated PCOS model mice. These findings further confirmed the role of Notch signaling in ER stress-enhanced expansion of COCs since ER stress is activated in granulosa cells of antral follicles in PCOS. A previous study demonstrated that ATF4, an UPR factor that was shown to mediate induction of Notch2 signaling by ER stress in the current study, plays a crucial role in COC expansion [44]. Our findings suggest that Notch signaling mediates the induction of COC expansion and its related genes by ER stress in PCOS ovaries.

There are several limitations of this study. One is that we did not examine whether COC expansion is enhanced in PCOS patients. It is quite difficult to measure the areas of COCs harvested at IVF since rapid manipulation of COCs is required in clinical settings for oocytes to have a good prognosis. Only one study compared the area of COCs harvested at IVF between patients with and without PCOS (n = 5, respectively), showing that the areas of COCs were significantly smaller in PCOS patients than in controls [45]. The authors also showed that mRNA expression of genes related to COC expansion was lower in COCs of PCOS patients than in those of controls. However, it is debatable whether mRNA expression of COC expansion-related genes in COCs harvested at IVF (i.e., more than 30 h after hCG administration) reflects the in vivo status of COC expansion. mRNA expression of these genes is induced shortly after hCG administration, peaks around 4–9 h after hCG administration, and decreases thereafter. Further study is needed to clarify whether COC expansion is dysregulated in humans in the same way as in model mice as shown in this study. Another limitation of our study is that the pathophysiological implication of enhanced COC expansion in PCOS was not determined. In particular, it is unclear whether this phenomenon is associated with ovulatory dysfunction, which is one of the main reproductive phenotypes of PCOS. COC expansion is a critical ovulatory event since animal models of cumulus matrix disruption frequently display impaired ovulation [46]. A recent study showed that prematurely ruptured dominant follicles in the natural cycles of infertile women often retain COCs, and the percentages of both immature and overmature COCs are higher among these unextruded COCs in postrupture follicles than among COCs harvested from prerupture follicles [47]. These findings indicate that abnormal maturation of COCs, in not only immature but also overmature COCs, is associated with a failure of COC extrusion, leading to ovulatory dysfunction. It remains to be elucidated whether enhanced COC expansion (i.e., overmature COCs) induced by ER stress via Notch signaling plays a causative role in ovulatory dysfunction in PCOS.

## 5. Conclusions

We showed that Notch2 signaling is activated in granulosa cells in PCOS. Notch signaling mediates ER stress-induced expansion of COCs. It remains to be elucidated whether aberrant COC expansion induced by the ER stress-Notch pathway is associated with ovulatory dysfunction in PCOS patients.

## Figures and Tables

**Table 1:** Characteristics of the patients who provided follicular fluid samples.

	Control (n = 12)	PCOS (n = 11)	*p*-Value
Age (years)	34.0 (26–40)	31.0 (24–37)	0.1236
BMI (kg/m^2^)	21.9 (19.7–25.4)	21.8 (18.1–26.4)	0.8916
LH (mIU/mL)	4.8 (2.1–5.6)	7.9 (5.9–15.4)	0.0002
FSH (mIU/mL)	7.2 (5.3–8.3)	6.5 (4.9–8.4)	0.1057
LH/FSH	0.64 (0.32–0.91)	1.28 (0.87–2.23)	<0.0001
AMH (ng/mL)	2.56 (1.51–6.73)	8.33 (5.44–24.63)	0.0010

Data are presented as median (range). BMI, body mass index; LH, luteinizing hormone; FSH, follicle-stimulating hormone; AMH, anti-Müllerian hormone.

**Table 2:** List of primers used for real-time qPCR.

Gene	Forward 5′–3′	Reverse 5′–3′
Notch2	CAACCGCAATGGAGGCTATG	GCGAAGGCACAATCATCAATGTT
Hey2	GCCCGCCCTTGTCAGTATC	CCAGGGTCGGTAAGGTTTATTG
Hes1	CCCAACGCAGTGTCACCTTC	TACAAAGGCGGCAATCCAATATG
ATF4	GGCTGGCTGTGGATGGGTTG	CTCCTGGACTAGGGGGGCAA
Areg	GTGGTGCTGTCGCTCTTGATA	CCCCAGAAAATGGTTCACGCT
Ereg	GTGATTCCATCATGTATCCCAGG	GCCATTCATGTCAGAGCTACACT
Tnfaip6	TTTCTCTTGCTATGGGAAGACAC	GAGCTTGTATTTGCCAGACCG
Has2	CTCTTTTGGACTGTATGGTGCC	AGGGTAGGTTAGCCTTTTCACA
COX2	CTGGCGCTCAGCCATACAG	CGCACTTATACTGGTCAAATCCC
GAPDH	TGGACCTGACCTGCCGTCTA	CTGCTTCACCACCTTCTTGA

## Data Availability

All of the data supporting the reported results are available in the manuscript.
